# Japan Society of Clinical Oncology Clinical Practice Guidelines 2017 for fertility preservation in childhood, adolescent, and young adult cancer patients: part 1

**DOI:** 10.1007/s10147-021-02081-w

**Published:** 2022-01-01

**Authors:** Miyuki Harada, Fuminori Kimura, Yasushi Takai, Takeshi Nakajima, Kimio Ushijima, Hiroaki Kobayashi, Toyomi Satoh, Akiko Tozawa, Kohei Sugimoto, Shigehira Saji, Chikako Shimizu, Kyoko Akiyama, Hiroko Bando, Akira Kuwahara, Tatsuro Furui, Hiroshi Okada, Koji Kawai, Nobuo Shinohara, Koichi Nagao, Michio Kitajima, Souichi Suenobu, Toshinori Soejima, Mitsuru Miyachi, Yoko Miyoshi, Akihiro Yoneda, Akihito Horie, Yasushi Ishida, Noriko Usui, Yoshinobu Kanda, Nobuharu Fujii, Makoto Endo, Robert Nakayama, Manabu Hoshi, Tsukasa Yonemoto, Chikako Kiyotani, Natsuko Okita, Eishi Baba, Manabu Muto, Iwaho Kikuchi, Ken-ichirou Morishige, Koichiro Tsugawa, Hiroyuki Nishiyama, Hajime Hosoi, Mitsune Tanimoto, Akira Kawai, Kazuhiko Sugiyama, Narikazu Boku, Masato Yonemura, Naoko Hayashi, Daisuke Aoki, Yutaka Osuga, Nao Suzuki

**Affiliations:** 1 Department of Obstetrics and Gynecology, Graduate School of Medicine, The University of Tokyo, 7-3-1, Hongo, Bunkyo, Tokyo, 113-8655 Japan; 2 Department of Obstetrics and Gynecology, Shiga University of Medical Science, Seta Tsukinowa-Cho Otsu, Shiga, 520-2192 Japan; 3 Department of Obstetrics and Gynecology Saitama Medical Center, Saitama Medical University, 1981 Kamoda, Kawagoe City, Saitama 350-3550 Japan; 4 Department of Endoscopy, Gastrointestinal Endoscopy Division, National Cancer Center Hospital, 5-1-1 Tsukiji, Chuo-ku, Tokyo, 104-0045 Japan; 5 Department of Obstetrics and Gynecology, Kurume University School of Medicine, 67 Asahi-machi, Kurume, Fukuoka 830-0011 Japan; 6 Department of Obstetrics and Gynecology, Graduate School of Medical and Dental Sciences, Kagoshima University, 8-35-1 Sakuragaoka, Kagoshima, 890-8520 Japan; 7 Department of Obstetrics and Gynecology, Faculty of Medicine, University of Tsukuba, Tennoudai, Tsukuba, Ibaraki 305-8575 Japan; 8 Department of Obstetrics and Gynecology, St. Marianna University School of Medicine, 2-16-1 Sugao, Miyamae, Kawasaki, Kanagawa 216-8511 Japan; 9 International Center for Reproductive Medicine, Dokkyo Medical University Saitama Medical Center, 2-1-50 Minamikoshigaya, Koshigaya, Saitama 343-8555 Japan; 10 Department of Medical Oncology, Fukushima Medical University, 1 Hikarigaoka, Fukushima-city, Fukushima 960-1295 Japan; 11 Department of Breast and Medical Oncology, Comprehensive Cancer Center, National Center for Global Health and Medicine, 1-21-1 Toyama, Shinjuku-ku, Tokyo, 162-8655 Japan; 12 Department of Breast and Endocrine Surgery, St. Marianna University School of Medicine, 2-16-1 Sugao, Miyamae, Kawasaki, Kanagawa 216-8511 Japan; 13 Department of Breast and Endocrine Surgery, Faculty of Medicine, University of Tsukuba, Tennoudai, Tsukuba, Ibaraki 305-8575 Japan; 14 Ladies Clinic Cosmos Kochi, 6-27, Sugiiru, Kochi, Kochi 780-0082 Japan; 15 Department of Obstetrics and Gynecology, Gifu University Graduate School of Medicine, 1-1, Yanagido, Gifu City, Gifu 501-1194 Japan; 16 Department of Urology, International University of Health and Welfare, 852, Hatakeda Narita, Chiba, 286-0124 Japan; 17 Department of Renal and Genitourinary Surgery, Hokkaido University Graduate School of Medicine, Kita 15Nishi 7, Kita-ku, Sapporo, Hokkaido 060-8638 Japan; 18 Department of Urology, Toho University Faculty of Medicine, 6-11-1, Omori-Nishi, Ota-ku, Tokyo, 143-8541 Japan; 19 Department of Obstetrics and Gynecology, Nagasaki University Graduate School of Biomedical Sciences, 1-12-4 Sakamoto, Nagasaki, 852-8523 Japan; 20 Division of General Pediatrics and Emergency Medicine, Department of Pediatrics, Oita University Faculty of Medicine, 1-1 Idaigaoka, Hasama, Yufu, Oita 879-5593 Japan; 21 Department of Radiation Oncology, Kobe Proton Center, 1-6-8, Minatojima-minamimachi, Chuo-ku, Kobe City, Hyogo 650-0047 Japan; 22 Department of Pediatrics, Graduate School of Medical Science, Kyoto Prefectural University of Medicine, 465 Kajii-cho, Hirokoji, Kamigyo-ku, Kyoto, 602-8566 Japan; 23 Department of Health and Nutrition, Faculty of Health and Nutrition, Osaka Shoin Women’s University, 4-2-26 Hishiya-nishi, Higashi-Osaka, Osaka 577-8550 Japan; 24 Division of Pediatric Surgical Oncology, National Cancer Center Hospital, 5-1-1 Tsukiji, Chuo-ku, Tokyo, 104-0045 Japan; 25 Department of Gynecology and Obstetrics, Kyoto University Graduate School of Medicine, 54 Kawahara-cho, Shogoin, Sakyoku, Kyoto, 606-8507 Japan; 26 Pediatric Medical Center, Ehime Prefectural Central Hospital, 83 Kasuga-machi, Matsuyama-city, Ehime 790-0024 Japan; 27 Division of Clinical Oncology and Hematology, Department of Internal Medicine, The Jikei University School of Medicine, 3-19-18 Nishi-Shinbashi, Minato-ku, Tokyo, 105-8461 Japan; 28 Division of Hematology, Department of Medicine, Jichi Medical University, 1-847 Amanuma, Omiya-ku, Saitama-city, Saitama 330-8503 Japan; 29 Division of Transfusion, Okayama University Hospital, 2-5-1 Shikata-cho, Kita-ku, Okayama, 700-8558 Japan; 30 Department of Orthopaedic Surgery, Kyushu University, Maidashi 3-1-1, Higashi-ku, Fukuoka, 812-8582 Japan; 31 Department of Orthopaedic Surgery, Keio University School of Medicine, 35 Shinanomachi, Shinjuku, Tokyo, 160-8582 Japan; 32 Department of Orthopedic Surgery, Osaka City University Graduate School of Medicine, 1-4-3 Asahi-Machi, Abeno-Ku, Osaka, 545-8585 Japan; 33 Division of Orthopedic Surgery, Chiba Cancer Center, 666-2 Nitona-cho, Chuo-ku, Chiba, 260-8717 Japan; 34 Children’s Cancer Center, National Center for Child Health and Development, 2-10-1 Okura, Setagaya-ku, Tokyo, 157-8535 Japan; 35 Department of Gastrointestinal Medical Oncology, National Cancer Center Hospital, 5-1-1 Tsukiji, Chuo-ku, Tokyo, 104-0045 Japan; 36 Department of Oncology and Social Medicine, Graduate School of Medical Sciences, Kyushu University, 3-1-1 Maidashi, Higashi-ku, Fukuoka, 812-8582 Japan; 37 Department of Therapeutic Oncology, Kyoto University Graduate School of Medicine, 54 Kawahara-cho, Shogoin, Sakyoku, Kyoto, 606-8507 Japan; 38 Department of Obstetrics and Gynecology, Medical Park Yokohama, 1-1-8, Sakuragi-cho, Yokohama, Kanagawa 231-0062 Japan; 39 Department of Urology, Faculty of Medicine, University of Tsukuba, Tennoudai, Tsukuba, Ibaraki 305-8575 Japan; 40 Department of Nursing, Doshisha Women’s College of Liberal Arts, Kodo, Kyotanabe City, Kyoto 610-0395 Japan; 41 Chugoku Central Hospital, 148-13, Kamiiwanari, Miyuki-cho, Fukuyama-city, Hiroshima 720-0001 Japan; 42 Department of Musculoskeletal Oncology and Rehabilitation Medicine, National Cancer Center Hospital, 5-1-1 Tsukiji, Chuo-ku, Tokyo, 104-0045 Japan; 43 Department of Clinical Oncology and Neuro-Oncology Program, Hiroshima University Hospital, 1-2-3 Kasumi, Minami-ku, Hiroshima, 734-8551 Japan; 44 Department of Medical Oncology and General Medicine, Institute of Medical Science, IMSUT Hospital, University of Tokyo, 4-6-1, Shirokanedai, Minato-ku, Tokyo, 108-8639 Japan; 45 Department of Pharmacy, National Cancer Center Hospital East, 6-5-1 Kashiwanoha, Kashiwa-shi, Chiba, 277-0882 Japan; 46 Graduate School of Nursing Science, St Luke’s International University, 10-1 Akashi-cho, Chuo-ku, Tokyo, 104-0044 Japan; 47 Department of Obstetrics and Gynecology, Keio University School of Medicine, 35 Shinanomachi, Shinjuku, Tokyo, 160-8582 Japan

**Keywords:** Practice guideline, Fertility preservation, Childhood, adolescent and young adult (CAYA)

## Abstract

In 2017, the Japan Society of Clinical Oncology (JSCO) published the JSCO Clinical Practice Guidelines 2017 for Fertility Preservation in Childhood, Adolescent, and Young Adult Cancer Patients. These were the first Japanese guidelines to address issues of oncofertility. In this field of medicine, sustained close cooperation between oncologists and reproductive specialists is essential from the diagnosis of cancer until many years after completion of cancer treatment. These JSCO guidelines were intended to guide multidisciplinary medical staff in considering the availability of fertility preservation options and to help them decide whether to provide fertility preservation to childhood, adolescent, and young adult cancer patients before treatment starts, with the ultimate goal of improving patient survivorship. The guidelines are presented as Parts 1 and 2. This article (Part 1) summarizes the goals of the guidelines and the methods used to develop them and provides an overview of fertility preservation across all oncology areas. It includes general remarks on the basic concepts surrounding fertility preservation and explanations of the impacts of cancer treatment on gonadal function by sex and treatment modality and of the options for protecting/preserving gonadal function and makes recommendations based on 4 clinical questions. Part 2 of these guidelines provides specific recommendations on fertility preservation in 8 types of cancer (gynecologic, breast, urologic, pediatric, hematologic, bone and soft tissue, brain, and digestive).

## Introduction

### Goal of the Japan Society of Clinical Oncology Guidelines

In childhood, adolescent, and young adult (CAYA) cancer patients, increasing attention is being paid to fertility preservation before the start of cancer treatment, with the aim to improve patients’ survivorship. However, the top priority in cancer patients is treating the cancer, and doing so without delay is a main principle of cancer care. As a consequence, depending on the type and stage of cancer, many CAYA cancer patients lose their ability to have a child in the future. Nevertheless, fertility could have been preserved in some cancer patients by more effective reproductive counseling before the start of cancer treatment. Major barriers to effective reproductive counseling of cancer patients include the lack of oncologists’ knowledge of reproductive medicine and the lack of their close cooperation with reproductive specialists. Thus, in the field of oncofertility, sustained close cooperation between oncologists and reproductive specialists is essential from the diagnosis of cancer until many years after completion of cancer treatment. Hence, the Japan Society of Clinical Oncology (JSCO) developed these Clinical Practice Guidelines 2017 for Fertility Preservation in CAYA Cancer Patients to guide multidisciplinary medical staff in considering the availability of fertility preservation options and to help them decide whether to provide fertility preservation to CAYA cancer patients before treatment starts, with the ultimate goal of improving patient survivorship.

### Methods used to develop the JSCO Guidelines

#### Outline

The Guideline Working Committee, which was established in November 2015, held a consensus meeting in October 2016. After hearing opinions from 23 scientific societies and 6 patient associations, the Guideline Working Committee wrote draft guidelines and submitted them to the Oncology Clinical Practice Guideline Review Committee for review with the Appraisal of Guidelines for Research & Evaluation (AGREE) II instrument. These procedures led to finalization of the guidelines in June 2017. The Guideline Working Committee consisted of experts from the 8 oncology areas addressed in these guidelines (gynecologic, breast, urologic, pediatric, hematologic, bone and soft tissue, brain, and digestive) and from reproductive, nursing, and pharmaceutical fields. While developing these guidelines, the Committee also received cooperation and support from the research project Development of the Infrastructure of Oncofertility in Japan (representative investigator: Yutaka Osuga), which was established by the Japan Agency for Medical Research and Development (AMED) in the fiscal year 2016.

Because oncofertility medicine lacks full evidence from randomized controlled trials (RCTs), these JSCO Guidelines are consensus based and not evidence based. The guidelines are presented as Parts 1 and 2. Part 1 summarizes the goals of the guidelines and the methods used to develop them and provides an overview of fertility preservation across all oncology areas. It includes general remarks on the basic concepts surrounding fertility preservation and explanations of the impacts of cancer treatment on gonadal function by sex and treatment modality and of the options for protecting/preserving gonadal function and makes recommendations based on 4 clinical questions. Part 2 presents specific aspects of fertility preservation in 8 types of cancer: gynecologic, breast, urologic, pediatric, hematologic, bone and soft tissue, brain, and digestive. The chapter on gynecologic cancers also discusses what reproductive support should be provided in view of the direct involvement of the female reproductive organs in this type of cancer, but the other chapters do not go beyond fertility preservation.

#### Literature search and data extraction and review

In cooperation with the Japan Medical Library Association, appropriate keywords were used to search the literature for articles published between January 2006 and November 2015. In addition, some articles published outside this period were retrieved manually if they were considered important by any member of the Guideline Working Committee. Also included in the review were some articles published before 2005 that were referenced in the American Society of Clinical Oncology (ASCO) 2006 Recommendations on Fertility Preservation in Cancer Patients [1]. All guidelines, reviews, and clinical statistical reports that were reviewed are listed in the References section. Evidence from each article was categorized as specified in Table 1.

**Table 1:** Categories of evidence levels

Category of evidence level	Description
Level I	Evidence from a systematic review or meta-analysis of randomized controlled trials (RCTs)
Level II	Evidence from 1 or more RCTs
Level III	Evidence from 1 or more non-randomized controlled studies
Level IVa	Evidence from 1 or more analytical epidemiology studies (cohort studies)
Level IVb	Evidence from 1 or more analytical epidemiology studies (case–control studies and/or cross-sectional studies)
Level V	Evidence from 1 or more descriptive studies (1 or more case reports and/or 1 or more case-series studies)
Level VI	Based on the opinion of experts (boards and/or individuals) without supportive patient data

The secondary sources referenced to develop the JSCO Guidelines included the ASCO 2006 Clinical Practice Guidelines [1] and 2013 update [2], the 2011 practical recommendations for fertility preservation in women from the FertiPROTEKT network [3], and the 2012 recommendations from the International Society for Fertility Preservation (ISFP) Practice Committee [4–6]. Other secondary sources referenced to develop the recommendations for individual clinical questions (CQs) are cited at the appropriate places under each CQ.

#### Development and grading of guideline recommendations

Drafts written by individual members of the Guideline Working Committee were reviewed by the field subcommittee and then cross-reviewed by other field subcommittees. The guideline recommendations and their grades were finalized by unanimous consent of the core members, i.e., the Guideline Working Committee chair, sub-chair, and supervisors (one oncologist and one reproductive specialist), and one representative member each from the nursing, pharmaceutical, and other fields. The definitions of recommendation grades used in these guidelines are shown in Table 2.

**Table 2:** Definitions of recommendation grades

Recommendation grade	Definition
A	Based on full scientific evidence, the approach is strongly recommended
B	Based on scientific evidence, the approach is recommended
C1	Despite the presence of limited scientific evidence, the approach is recommended
C2	Because of the paucity of scientific evidence, the approach is not recommended
D	Based on scientific evidence for its non-efficacy or harm(s), the approach is not recommended

## Fertility preservation in CAYA cancer patients in general

### General remarks

Improvements in cancer treatment for CAYA patients have increased the number of cancer survivors. On the other hand, certain types of cancer treatment are known to affect fertility, and the accumulated evidence indicates that cancer survivors have a risk of both infertility and insufficient sex hormone secretion. Because awareness of these risks has increased, more efforts are currently made to protect or preserve germ cells in cancer patients before they receive treatment. However, much remains to be clarified regarding fertility preservation in cancer patients, referred to as oncofertility, including the impacts of oncofertility interventions on the prognosis of the underlying malignancy, the future possibility of achieving pregnancies with the cryopreserved germ cells or gonadal tissues, and the outcomes of pregnancies achieved by such measures. Hence, health care providers should fully understand the current standards of oncofertility medicine and the associated ethical problems. Before administering any fertility preservation treatment, health care providers should assess the suitability of such treatment by considering the impacts of various modalities of cancer treatment on gonadal function and the current status of the cancer; to do so, they require full knowledge of the currently available fertility preservation options. Health care providers should also make efforts to understand issues around the fertility of patients with hereditary cancer. Physicians and other health care professionals who care for cancer patients at or before reproductive age should provide them and their family members with reproductive counseling and, if necessary, refer them to reproductive specialists to help them make decisions regarding fertility preservation. The decision made by the patient and their family members after counseling by reproductive specialists should be respected, unless the decision is expected to adversely affect the patient’s cancer treatment. Even patients who state that they are not interested in having a child should be informed that they have to receive endocrine follow-up after cancer treatment to improve cancer survivorship care.

### Impacts of cancer treatment on gonadal function by sex and treatment modality

Cancer treatment is provided by various modalities, including surgery, chemotherapy, radiotherapy, and endocrine therapy. As summarized in Table 3, different modalities of cancer treatment can have different impacts on gonadal function by sex because of the sex-related differences in gametogenesis, more specifically, in the responsible organs, their location in the body, the processes involved, and the form of storage. For additional information, readers can also refer to the relevant ASCO guidelines [2], which contain a list of treatment protocols with a high, intermediate, low, very low, or unknown risk of infertility in each sex, stratified by target cancer and by other factors affecting fertility (e.g., patient population and drug dose).

**Table 3:** Impacts of cancer treatment on gonadal function by sex and treatment modality

Impacts in female patients	
Surgery	Ovarian and other pelvic organ surgery may reduce the number of ovarian follicles (each of which is a cellular aggregation containing an immature oocyte surrounded by its encasing cells, such as granulosa cells and theca cells; the maturation of follicles is associated with the maturation of oocytes) and suppress ovarian sex hormone production, leading to ovarian failure
Chemotherapy	Many anticancer agents inhibit the growth of ovarian follicles, causing temporary, i.e., reversible, amenorrhea. On the other hand, alkylating agents (e.g., cyclophosphamide and busulfan) and platinum analogs (e.g., cisplatin) are highly gonadotoxic and can reduce the number of oocytes. Treatment with any such agent at a high cumulative dose can cause permanent loss of oocytes soon after treatment and reduce ovarian hormone production
Radiotherapy	Ovarian radiation can reduce the number of oocytes and impair ovarian function. Radiation at a high cumulative dose can cause permanent loss of oocytes soon after treatment and reduce ovarian hormone production. Hypothalamic or pituitary radiation may impair ovulation
Impacts in males	
Surgery	Testicular surgery may interfere with spermatogenesis, testicular hormone production, and spermatozoa transportation, leading to testicular failure
Chemotherapy	Alkylating agents (e.g., cyclophosphamide, ifosfamide, busulfan, and procarbazine) reduce the number of spermatogonia. Treatment with any such agent at a high cumulative dose can cause permanent impairment of spermatogenesis soon after treatment
Radiotherapy	Testicular radiation can reduce the number of spermatogonia. Radiation at a high cumulative dose can cause permanent impairment of spermatogenesis soon after treatment. Hypothalamic or pituitary radiation may impair spermatogenesis and/or hormone production
Impacts in both sexes	
Interferon-α and tyrosine kinase inhibitors can induce thyroid function abnormalities	

#### Impacts of cancer treatment in female patients

Uterine or ovarian surgery is expected to impair ovarian function by interfering with perfusion of the uterus or ovaries or both. Ovarian surgery has been reported to reduce the serum level of anti-Müllerian hormone (AMH), a marker of ovarian reserve that is generally correlated with the number of oocytes contained in the ovaries [7]. Bilateral oophorectomy results in complete loss of ovarian function, whereas unilateral oophorectomy, partial ovarian resection, and their combination reduce the number of oocytes contained in ovarian tissue.

Some anticancer agents have a considerable impact on oocytes and ovarian function, whereas others have little impact on them [8]. Even those agents that impair oocytes and/or ovarian function act on different cells: some directly affect oocytes contained in the ovary, whereas others affect cells such as granulosa cells, which encase oocytes [8]. Granulosa cells actively divide and are therefore susceptible to the toxicity of many anticancer agents. Agents that are toxic to granulosa cells inhibit the maturation of follicles, each of which is a cellular aggregation containing an immature oocyte surrounded by its encasing cells, including granulosa cells, as well as theca cells; the maturation of follicles is associated with the maturation of oocytes, and mature follicles are responsible for the production of female sex hormones. Thus, treatment with anticancer agents can temporarily reduce the production of female sex hormones and thereby induce amenorrhea [8]. Chemotherapy-induced amenorrhea is often reversible after completion of chemotherapy if the chemotherapy is primarily toxic to mature follicles. However, certain chemotherapeutic agents reduce the number of oocytes contained in the ovary and, consequently, can potentially cause lifelong impairment of ovarian function. Alkylating agents, such as cyclophosphamide and busulfan, and platinum analogs, such as cisplatin, are representative of this class of chemotherapy [9–14]. This type of chemotherapy-induced amenorrhea has been reported to occur in 30% to 76% of patients receiving such chemotherapy [8]. As the cumulative dose of such anticancer agents increases, the number of primordial follicles decreases [14]. Depending on the cumulative dose of these agents, permanent ovarian failure (loss of oocytes and reduced hormone production) may occur soon after treatment. A formula has been developed that uses the cumulative cyclophosphamide equivalent dose of an alkylating agent to predict the probability that the agent will reduce ovarian function [14]. Susceptibility to the gonadotoxicity of these agents also depends on the age at treatment, with patients treated at higher ages being more likely to develop premature ovarian insufficiency (POI) after treatment [8].

Ovarian radiation, not only direct but also scatter radiation, also reduces the number of primordial follicles contained in the ovary. A higher cumulative radiation dose causes greater ovarian impairment. However, one study suggested that even radiation at a dose of only 2 Gy can reduce the number of primordial follicles contained in the ovary [15]. Another study showed that radiation at a dose of 20.4 Gy at birth or at doses of 14.3 Gy or more at age 30 leads to a complete loss of ovarian function [16]. Thus, patients irradiated at higher ages are more susceptible to the toxicity of radiation and more likely to develop POI [16–18]. Hypothalamic or pituitary radiation can impair gonadotropin secretion and thereby cause ovarian failure, and radiation at 35–40 Gy is known to lead to insufficient gonadotropin secretion. Hypothalamic or pituitary radiation is not directly toxic to ovarian tissue but impairs ovarian function via a centrally mediated mechanism [19].

#### Impacts of cancer treatment in male patients

Testicular surgery, which is primarily indicated for testicular tumors, may interfere with spermatogenesis, testicular hormone production, and spermatozoa transportation. An observational study found that 85% of patients with a testicular tumor who underwent unilateral orchiectomy with no adjuvant chemotherapy or radiotherapy were able to have a child during the 11 year follow-up, suggesting that this surgical procedure has no substantial impact on male fertility [20]. However, bilateral orchiectomy leads to a complete loss of spermatogenesis and testicular hormone production.

Some anticancer agents have considerable effects on spermatogenesis and testicular function in that they affect spermatogonia (the cellular origin of spermatogenesis) and Leydig cells (the cells that encase spermatogonia and produce male sex hormones) [21]. Spermatogonia actively divide and thus are more susceptible to the toxicity of anticancer agents than Leydig cells, which do not actively divide [22]. Hence, even patients who remain able to produce male sex hormones after receiving chemotherapy may have impaired spermatogenesis and azoospermia [22]. Even if spermatogenesis stops due to cancer treatment, it may resume several years after completion of chemotherapy [22]. Alkylating agents (e.g., cyclophosphamide, ifosfamide, busulfan, and procarbazine) and platinum analogs (e.g., cisplatin) reduce the number of spermatogonia [23–28]. Treatment with any such agent at a high cumulative dose can cause permanent impairment of spermatogenesis soon after treatment.

Testicular radiation also affects spermatogonia and Leydig cells. Similar to chemotherapy, radiotherapy is more likely to impair spermatogenesis than testicular hormone production because spermatogonia are more susceptible than Leydig cells to the effects of radiation [29]. A 11 year follow-up study showed reduced fertility after radiation at a dose of 7.5 Gy or more in patients receiving radiotherapy of the testis or surrounding tissue at age 20 years or younger as compared with their healthy siblings [28]. The study also found that temporary oligospermia and azoospermia can occur after radiation at doses of 0.10 Gy and 0.35 Gy, respectively [30]. Some reports suggest a risk of permanent azoospermia after radiation at a dose of 2–4 Gy or more [30–32]. The risk of azoospermia has been stratified according to testicular radiation dose and degree of sexual maturity [17].

Hypothalamic or pituitary radiation may impair gonadotropin secretion, thereby causing testicular failure [33], and radiation at 35–40 Gy can cause insufficient gonadotropin secretion [2].

#### Impacts of cancer treatment in both sexes

Some cancer treatments can affect fertility in both female and male patients. Interferon (IFN)-α frequently induces the formation of anti-thyroid autoantibodies, causing thyroid function abnormalities [34, 35]. In addition, tyrosine kinase inhibitors—a class of anticancer agent that inhibit a group of key enzymes involved in the proliferation, migration, and invasion of tumor cells—can induce hypothyroidism, with the probability of such an effect ranging from 32 to 85% [34–36].

### Options to protect/preserve gonadal function

The currently available options to protect/preserve gonadal function include pharmacological gonad protection and gonad transposition, which aims to leave intact germ cells in the body; and germ cell harvesting and cryopreservation before treatment. Gonad transposition is discussed in this section, and germ cell harvesting and cryopreservation are addressed in more detail in CQ2 and CQ3 below. However, pharmacological gonad protection with a gonadotropin releasing hormone (GnRH) agonist is discussed in the chapter on breast cancer in Part 2 of these guidelines.

Gonad transposition is offered when pelvic irradiation is performed as a cancer treatment. As a fertility preservation option in female patients, ovarian transposition (oophoropexy) may be considered before radiation for a primary or metastatic lesion of colon cancer, malignant lymphoma, rhabdomyosarcoma, Ewing sarcoma, or any other cancer within the pelvis near the ovaries. Radiotherapy for these cancers usually uses a dose of 14–60 Gy, although the dose required depends on the histological type of cancer. Ovarian radiation at such a dose can reduce the number of oocytes in the ovaries [37]. Therefore, if the disease status permits, oophoropexy should be considered during tumor resection or before the start of radiation. Extrapelvic, cranial lateral transposition of the ovaries is the most commonly used procedure, although medial transposition of the ovaries has also been reported in the treatment of malignant lymphoma that involves lymph nodes around large vessels [37]. To preserve ovarian function, the ovaries should be moved to and secured at a position as distant as possible from the point of radiation [38], although even this technique does not always protect the ovaries [37]. In male patients, a case report described testicular transposition in which a testis was moved to the contralateral scrotum to prevent its exposure to radiation [39].

## Clinical questions

This part of the guidelines addresses 4 important CQs regarding counseling of CAYA cancer patients and available assisted reproductive technology (ART) interventions. Recommendations are provided for each CQ, and the recommendation grade is provided in parentheses at the end of each recommendation.

### CQ1: how should CAYA cancer patients and/or their families be counseled about fertility?

#### Recommendation 1

1-1. Health care providers caring for CAYA cancer patients should prioritize cancer treatment over other interventions. (No Recommendation Grade).
1-2. Health care providers caring for CAYA cancer patients should inform them and/or their families about the possibility of infertility at reproductive ages after certain cancer treatments and should provide them with other relevant information. (No Recommendation Grade).
1-3. Health care providers caring for CAYA cancer patients should refer patients and their families to reproductive specialists as early as possible if the patients and/or their families are interested in counseling on fertility preservation. (No Recommendation Grade).
1-4. In close cooperation with reproductive specialists, health care providers caring for CAYA cancer patients should consider the availability of fertility preservation options and the optimal timing for each patient to undergo any intervention to preserve fertility. (No Recommendation Grade)

#### Explanations

The above recommendations indicate how health care providers caring for CAYA cancer patients should approach the topic of fertility preservation in their patients. No scientific evidence is available that definitely supports any recommendation because it is ethically difficult to conduct appropriate clinical studies to address this issue. However, a consensus was reached on several important points. Therefore, we decided to assign “no recommendation grade” to the above-mentioned recommendations.

Cancer treatments can have adverse effects on the reproductive/endocrine function of CAYA cancer patients. Because recent advancements in cancer therapy have increased the number of long-term survivors of cancer, one of the greatest concerns for CAYA cancer patients is how to maintain their gonadal function and preserve fertility after cancer-directed therapy [1, 40–43]. In recent years, several national and international guidelines have been issued for fertility preservation in cancer patients, including the ASCO 2006 Guidelines, which recommend that health care providers caring for cancer patients should inform them before treatment starts about the possibility of infertility, consider using any fertility preservation option available for eligible patients and refer such patients and/or their families to reproductive specialists [1]. The National Comprehensive Cancer Network Guidelines Insights on Adolescent and Young Adult (AYA) Oncology suggest that fertility preservation should be an essential part in the treatment of AYA patients (aged 15–39 years) with cancer. The Guidelines recommend that health care providers should discuss fertility preservation with all cancer patients before the start of treatment and refer AYA cancer patients who are eligible for or interested in fertility preservation interventions to reproductive specialists within 24 h after reproductive counseling [44]. If the scheduled cancer treatment is expected to abolish gonadal function or fertility, options for preserving future fertility should be discussed with patients as early as possible before the start of treatment [1–3, 41, 43]. With the consent of their oncologists, young cancer patients should receive concrete and accurate information on fertility preservation methods from reproductive specialists [1–3, 40]. Providing fertility preservation counseling to young cancer patients before the start of cancer treatment is thought to be an important issue that should be addressed generally in cancer treatment [42]. If possible, cancer patients should complete any fertility preservation intervention before the start of a cancer treatment that is expected to impair their gonadal function [2]. From an ethical perspective, it remains controversial how patients with cancer who are at risk of recurrence or at high risk of mortality should be informed about the possibility of fertility loss [45].

Despite increasing awareness about the importance of fertility preservation in CAYA cancer patients, many such patients still remain uninformed about fertility preservation before treatment starts [42, 46]. The underlying causes of this uninformed status of cancer patients include (i) oncologists’ lack of time for counseling patients about fertility preservation; (ii) oncologists’ lack of knowledge about fertility preservation; iii) oncologists’ unwillingness to discuss fertility and sexuality with CAYA cancer patients and/or their families; (iv) too low or high ages of patients and presence or absence of partners; (v) oncologists’ difficulties to collect information about fertility preservation and their unwillingness to communicate with patients and/or their families about issues that may result in a delay of cancer treatment or make them feel anxious about cancer treatment; and (vi) anticipated poor prognosis of patients [42, 46, 47]. A survey of 94 Irish oncologists (28 clinical oncologists, 32 hematologic oncologists, and 34 breast physicians) regarding their awareness of fertility preservation approaches in young cancer patients revealed a lack of knowledge about reproductive medicine and suggested that concerns about delaying cancer-directed therapy, anticipated poor prognosis of cancer, and a disease status (e.g., hormone receptor-positive breast cancer) might underlie the inadequate reproductive counseling of young cancer patients [48]. Another survey in 843 Japanese oncologists specialized in breast cancer care regarding their awareness of fertility preservation in young patients with breast cancer revealed that concerns about disease recurrence, lack of cooperation with reproductive specialists, and limited time to spare before the start of cancer-directed therapy might contribute to the lack of information about fertility preservation provided to patients [49].

Health care providers, including those caring for cancer patients, should choose optimal fertility preservation approaches on the basis of an overall assessment of the risk of infertility resulting from cancer treatment, the prognosis of the cancer, the risks associated with delaying initiation of cancer treatment, the impacts of future conception on the risk of cancer recurrence, and the potential impacts of hormonal manipulation on the biology of the cancer [2, 41]. However, oncologists should give first priority to providing cancer treatment, and counseling about infertility risk and fertility preservation approaches should not have any adverse effect on the outcome of cancer treatment or delay the start of treatment [42, 50]. Thus, oncologists should not take a fertility preservation approach that may reduce the response to cancer treatment [3]. Within a limited time before cancer treatment starts, CAYA cancer patients and/or their families should be given the maximum opportunity to receive information about fertility preservation that allows them to make their own decisions; nevertheless, cancer-directed therapy should be prioritized [51].

### CQ2: what assisted reproductive technology (ART) interventions are recommended for female cancer patients?

#### Recommendation 2

2-1. Embryo (fertilized oocyte) cryopreservation is recommended for female patients who have a male partner. (Recommendation Grade B).
2-2. Unfertilized oocyte cryopreservation may be considered for female patients who do not have a male partner. (Recommendation Grade C1).
2-3. Ovarian tissue cryopreservation, although it remains experimental, may be considered in centers with the necessary expertise for female patients who do or do not have a male partner if there is a need for urgent cancer-directed therapy, i.e., if there is limited time for embryo (fertilized oocyte) or unfertilized oocyte cryopreservation or if ovulation induction for oocyte harvesting is difficult (e.g., in prepubertal females). (Recommendation Grade C1)

#### Explanations

ART for infertile persons appears to have good safety and efficacy. ART is also important in the field of oncofertility, but little evidence is available about the optimal ART method for inducing ovulation or the effects of ART on maternal/neonatal health. There is a need to discuss the ethical and social problems relevant to the application of ART in the field oncofertility, to follow up and analyze cancer patients who have undergone ART treatments and to develop guidelines for ART treatments in cancer survivors. Female cancer patients should be counseled on an individualized basis; they should be fully informed that all fertility preservation methods available for female patients are associated with a greater physical burden than sperm cryopreservation for male patients and that these methods are not always successful. Table 4 provides an overview of fertility preservation methods available for female cancer patients. This section discusses these methods in more detail below and also addresses the acceptable time to consider pregnancy after completion of cancer chemotherapy and the optimal timing of oocyte/ovarian tissue harvesting. To develop recommendations in an attempt to answer this CQ, we referenced the secondary sources cited in the Introduction—sources that we also referenced for the whole guidelines—and the articles cited in this section, as well as references [52–56].

**Table 4:** Fertility preservation options available for female cancer patients

	Embryo (fertilized oocyte) cryopreservation	Unfertilized oocyte cryopreservation	Ovarian tissue cryopreservation
Eligible malignancies	Leukemia, breast cancer, lymphoma, digestive system cancer, gynecological cancer, malignant melanoma, germ cell tumor, brain tumor, sarcoma, etc	Leukemia, breast cancer, lymphoma, digestive system cancer, gynecological cancer, malignant melanoma, germ cell tumor, brain tumor, sarcoma, etc	Breast cancer, lymphoma, etc. (in cases where auto-grafting is considered)
Eligible ages^a^	16–45 years	16–40 years	0–40 years
In women with or without a partner	With a partner	Without a partner	With or without a partner
Time required for intervention	2–8 weeks	2–8 weeks	1–2 weeks
Method for freezing	Vitrification	Vitrification	Slow freezing or vitrification
Oocyte viability after thawing	≥ 95%–99%	≥ 90%	≥ 90%^c^
No. of successful deliveries^b^	Innumerable	≥ 6,000	≥ 60
Advantages/disadvantages	Pregnancy rate per embryo transfer: 30–35%	Pregnancy rate per oocyte retrieval: 4.5–12%	Can cryopreserve many oocytes; risk of minimal residual disease; low efficiency of ovarian follicle survival

^a^Eligible ages vary among clinics

^b^According to data published up to and including November 2015

^c^Not conclusive

##### Embryo cryopreservation

Embryo cryopreservation is an ART treatment that appears to have good efficacy and safety and is recommended as an effective oncofertility treatment option by the American Society for Reproductive Medicine (ASRM) [41], ASCO [2], and ISFP [4]. However, very limited evidence is available to support the efficacy and safety of embryo cryopreservation as an oncofertility treatment option.

To date, one group of investigators has reported on the use of embryo cryopreservation as an oncofertility treatment option: Oktay et al. [57] cryopreserved embryos produced by in vitro fertilization (IVF) of oocytes from 131 patients with breast cancer and transferred 81 thawed embryos to 33 patients in 40 cycles. These therapies resulted in 25 live births (30.9% per embryo transferred) in 18 cycles (45.0% per cycle of transfer). This live birth rate per embryo transferred was similar to that reported for infertile women undergoing oocyte harvesting at matched ages in the US general population (38.2%) [57]. According to another report from the same group [58], no significant difference was found in cancer recurrence or survival rate among 337 eligible patients with breast cancer between those who did (*n* = 120) and those who did not (*n* = 217) undergo any intervention to preserve fertility (embryo or unfertilized oocyte cryopreservation).

Oocyte harvesting for cryopreservation of embryos or unfertilized oocytes can have various complications. Oocyte harvesting is routinely performed by transvaginal puncture under ultrasound guidance and therefore, in case of injury, may cause hemorrhage from the vaginal wall or any intrapelvic vessel. It may even injure other organs, such as the intestine or the urinary bladder. Transvaginal puncture may induce pelvic peritonitis attributable to vaginal bacteria. If controlled ovarian stimulation is performed prior to oocyte harvesting, it is associated with the risks of ovarian hyperstimulation syndrome and thrombosis. Although embryo (fertilized oocyte) cryopreservation is a fertility preservation method of choice for female patients who have a male partner, unfertilized oocyte cryopreservation should remain an option for such patients because some circumstances do not permit sperm harvesting from the male partner.

##### Unfertilized oocyte cryopreservation

As discussed below, unfertilized oocyte cryopreservation has been established technically. However, little evidence is available to support its efficacy and safety as an oncofertility treatment option.

The ASRM Guidelines [59] state that unfertilized oocyte cryopreservation is no longer experimental but is a practicable technique with established efficacy and safety because similar fertilization and pregnancy rates have been achieved with vitrified/thawed oocytes versus fresh oocytes and because there is no increase in the frequency of chromosomal aberrations, congenital anomalies, or developmental disorders in neonates born with ART that uses cryopreserved/thawed oocytes. The ASRM Guidelines also suggest that unfertilized oocyte cryopreservation is an effective oncofertility treatment option and should be performed with appropriate counseling. The FertiPROTEKT oncofertility network, which comprises 101 centers in 3 countries (including Germany), has also developed guidelines on the indications for unfertilized oocyte cryopreservation [3], and the UK National Institute for Health and Clinical Excellence has published guidelines that suggest that cryopreservation of unfertilized oocytes is a useful technology for reproductive medicine [60]. In Japan, the Japan Society for Reproductive Medicine and the Japan Society of Obstetrics and Gynecology have published joint guidelines on medical indications for unfertilized oocyte cryopreservation and ovarian tissue cryopreservation.

Significantly better reproductive outcomes have been achieved with vitrified oocytes than with slowly frozen oocytes [61]. As suggested by a meta-analysis of RCTs [62], similar fertility and pregnancy rates may be achieved with vitrified oocytes as with fresh oocytes, with a pregnancy rate of 4.5–12% per thawed oocyte [59]. A comparison of neonatal health showed no difference in mean birth weight or frequency of congenital anomalies between neonates born from vitrified oocytes and those born from fresh oocytes [63]. However, the majority of oocytes included in the above-mentioned studies were harvested from young donors or generally infertile women with adequate ovarian reserve. Therefore, further studies are needed to determine whether these results can be generalized to all age groups and patients at all reproductive clinics and even to cancer survivors.

##### Ovarian tissue cryopreservation

Very limited evidence is available to support the efficacy and safety of ovarian tissue cryopreservation as an oncofertility treatment option.

Controlled ovarian stimulation with a stimulating agent is almost essential for oocyte harvesting for IVF and/or cryopreservation, but this procedure may delay the initiation of cancer-directed therapy. In addition, only a limited number, i.e., several to about 20, fertilized or unfertilized oocytes can be obtained. On the other hand, ovarian tissue cryopreservation, which does not require ovarian stimulation, can be performed immediately via a less invasive laparoscopic approach and also be performed in prepubertal females. However, in current procedures a substantial portion of thousands of oocytes contained in the ovarian cortex are lost during freezing/thawing and transplantation, and preventing this loss remains a challenge.

Slow freezing uses a programmed freezer to slowly freeze tissue samples. At least 60 successful pregnancies/deliveries have been reported after ovarian tissue cryopreservation, mostly with slow freezing [64]. Vitrification can be done quickly without using a programmed freezer, which has contributed to its widespread use in clinical practice for fertilized or unfertilized oocyte cryopreservation, although its use for human ovarian tissue cryopreservation has long been experimental. Recently, a vitrification technique for ovarian tissue cryopreservation was developed in Japan that enables bedside cryopreservation in an operative room within 1 h after tissue harvesting. This technique is becoming more popular, primarily in Japan, with several successful deliveries from patients with POI reported after transplantation of ovarian tissue vitrified by this technique [65].

To date, auto-grafting is the only way to use cryopreserved ovarian tissue in practice. After grafting, it usually takes 4–5 months to resume follicle growth and recover ovarian function. A 2014 review identified 35 live births from 121 patients (28.9%) undergoing auto-grafting of frozen–thawed ovarian tissue [66]. Orthotopic grafting is performed onto the residual ovarian section or the retroperitoneum near the site where the ovary was present, whereas heterotopic grafting is performed into the rectus abdominis muscle or the forearm. Heterotopic grafting is advantageous over orthotopic grafting in that it requires a simpler surgical procedure, allows easier access to a malignant tumor recurring within the graft and is an effective alternative if orthotopic grafting is prevented by prior radiotherapy. Only orthotopic ovarian tissue grafting had given rise to live births until a report in 2014 of successful deliveries with the aid of ART treatment after heterotopic ovarian tissue grafting [67].

The use of auto-grafting of cryopreserved ovarian tissue may be limited by the risk of malignant cells contaminating the tissue (minimal residual disease). Although evidence is insufficient to rule out this possibility, no cases of cancer recurrence due to re-transfer of tumor cells have been reported after auto-grafting of cryopreserved ovarian tissue. Therefore, it is likely that this fertility preservation method can be used safely if its indication is carefully considered with respect to the type and stage of cancer. Another review suggested that ovarian tissue cryopreservation may be primarily indicated for fertility preservation in patients with Hodgkin lymphoma, non-Hodgkin lymphoma, and breast cancer [68]. Before frozen ovarian tissue is thawed and grafted, patients should be fully informed about the benefits and risks of the procedure, and aliquots of the tissue should be tested for contamination of malignant cells by histopathological examination, immunostaining, and (if possible) polymerase chain reaction. At present, the most reliable way to determine whether malignant cells are present in the graft is to observe animals xenografted with the patient ovarian tissue for at least 20 weeks [68].

##### Acceptable time to consider pregnancy after completion of cancer chemotherapy and optimal timing of oocyte/ovarian tissue harvesting for cryopreservation

Developing human embryos/fetuses are susceptible to teratogenicity during the period of fetal organogenesis, i.e., at 2–8 weeks after fertilization (gestation weeks 4–10), especially at 3–5 weeks after fertilization (gestation weeks 5–7) [69]. Although little evidence is available regarding the acceptable time to consider pregnancy after completion of cancer chemotherapy, contraception is generally recommended for 4–6 months after completion of chemotherapy to ensure complete elimination of the potentially fetotoxic drug(s) before pregnancy and to take into account the risk of cancer recurrence immediately after completion of treatment [70].

In mice, treatment with cyclophosphamide 6 weeks before IVF caused a significant decrease in the rates of fertilization and embryo development and a significant increase in the frequency of aneuploid embryos compared with the control [71]. The frequency of congenital anomalies is generally believed to be unlikely to increase in babies born to cancer survivors, although there have been several reports of an increase of abortions/premature deliveries and low birth weight infants associated with previous cancer treatment [72]. In addition, no definitive evidence suggests any adverse impacts of oocyte or ovarian tissue harvesting immediately after completion of chemotherapy on outcomes of the next generation. Nonetheless, patients should be fully informed about the benefits and risks of fertility preservation treatment beforehand and be carefully managed and followed up after receiving any such intervention.

### CQ3: which ART interventions are recommended for male cancer patients?

#### Recommendation 3

Male cancer patients should be counseled about the following fertility preservation options *before* receiving cancer-directed therapy:

3-1. Sperm cryopreservation is recommended and should be performed before initiation of chemotherapy. (Recommendation Grade B).
3-2. Nerve-sparing surgery is recommended if surgery is likely to cause erectile/ejaculatory dysfunction. (Recommendation Grade B)

Male cancer patients should be counseled about the following fertility preservation options *after* receiving cancer-directed therapy:

3-3. Testicular sperm extraction (TESE) may be considered for the management of chemotherapy-induced azoospermia. (Recommendation Grade C1).
3-4. Hormone replacement therapy is recommended for acquired pituitary hypogonadotropic hypogonadism. (Recommendation Grade B)

#### Explanations

Mechanisms involved in impaired male fertility resulting from cancer treatment include chemotherapy-induced impairment of spermatogenesis; impairment of spermatogenesis and/or erectile/ejaculatory dysfunction associated with hypothalamic–pituitary–gonadal axis endocrine disorders; erectile/ejaculatory dysfunction due to a nervous system disorder after surgery involving the inferior hypogastric plexus; and resection of male gonads, including the testes and prostate. Some case reports have described patients with temporary azoospermia after cancer treatment in whom spontaneous recovery of spermatogenesis or TESE followed by ART eventually enabled them to have a child. Although these JSCO guidelines address fertility preservation in CAYA patients with cancer, it should be noted that male cancer patients over 40 years of age should also be counseled about fertility preservation if their partners are of reproductive age. This section provides a brief review of the available fertility preservation options for male cancer patients that patients should be informed about before receiving cancer-directed therapy and after completion of treatment. It also discusses the acceptable time to consider pregnancy after completion of cancer chemotherapy and the optimal timing of sperm harvesting for cryopreservation. To develop recommendations in an attempt to answer this CQ, we referenced the secondary sources cited in the Introduction—sources that we also referenced for the whole guidelines—and the articles cited in this section, as well as references [52, 55, 56].

##### Fertility preservation options that male cancer patients should be informed about before receiving cancer-directed therapy

*Sperm cryopreservation before chemotherapy* Sperm cryopreservation was developed several decades ago as an intervention for infertility treatment and now has an established efficacy and safety profile. It has also been used as a fertility preservation option for male cancer patients and is recommended before treatment starts for male cancer patients who are at risk of developing azoospermia (e.g., those scheduled to undergo chemotherapy or bilateral orchiectomy) and interested in having a child [1, 73].

Masturbation is the most commonly used method to collect sperm. Cancer per se frequently impairs spermatogenesis, resulting in oligospermia. For men who are unable to masturbate, alternative methods for collecting sperm can be used, including urinary sperm retrieval in men with retrograde ejaculation and induction of ejaculation by penile vibratory stimulus and electroejaculation. Sperm may be obtained even from patients with azoospermia (no spermatozoa in ejaculated seminal fluid) using oncological TESE (which is also referred to as onco-TESE).

A systematic review of 30 studies of reproductive outcomes with cryopreserved sperm in a total of 11,798 male cancer patients [74] showed that the overall rate of sperm utilization was as low as 8% [95% confidence interval (CI), 8–9%] and that the overall rate of live births with cryopreserved sperm was 49% (95% CI 44–53%). Thus, the partners of very few of the men who underwent sperm cryopreservation had live babies. However, the low rate (16%; 95% CI 15–17%) of disposal of sperm specimens and the significant positive correlation between the duration of follow-up and the sperm utilization rate suggest that the rate of utilization of cryopreserved sperm may increase progressively during follow-up after cancer treatment. As an ART treatment with cryopreserved sperm, intracytoplasmic sperm injection (ICSI) is more likely to result in live births than intrauterine insemination (IUI) or IVF [75, 76]. Therefore, in recent years many reproductive clinics have preferentially performed ICSI. The above-mentioned systematic review also showed a similar frequency of congenital anomalies in babies born after use of cryopreserved sperm from male cancer patients (4%; 95% CI 1–11%) and in babies born in the general population [74]. Thus, definitive evidence currently supports the efficacy and safety of sperm cryopreservation as an oncofertility treatment option. On the other hand, the safety of using sperm harvested after the start of cancer chemotherapy has not been established, and no consensus has been reached regarding the suitability of cryopreserving sperm harvested from male cancer patients after the start of cancer chemotherapy.

*Testicular tissue cryopreservation before cancer treatment in prepubertal boys* As mentioned above, cryopreservation of ejaculated spermatozoa is an established oncofertility treatment option for postpubertal male patients. In prepubertal boys, testicular tissue cryopreservation has been attempted as an intervention to preserve fertility, but it has only resulted in live born babies in the partners of some patients who had spermatozoa or spermatids in the testicular tissue. Various approaches have been developed and tested for their ability to stimulate immature testes to induce the differentiation of spermatogonia toward spermatids or spermatozoa, but to date no effective method has been established in humans [77].

*Fertility-preserving surgery and fertility preservation treatment for the prophylaxis or management of erectile/ejaculatory dysfunction* The most serious postoperative complications that may cause infertility are retrograde ejaculation after retroperitoneal lymph node dissection for the management of testicular cancer and erectile/ejaculatory dysfunction after radical surgery for colon cancer that involves the inferior hypogastric plexus. To prevent these nerve injuries, nerve-sparing surgery should be performed, if possible. However, a nerve-sparing procedure may be contraindicated because of the need to enhance the treatment success of surgery. If erectile/ejaculatory dysfunction is inevitable after surgery, TESE may enable sperm retrieval. Urinary sperm retrieval is indicated for men with retrograde ejaculation, and sperm retrieved from the urinary bladder may be used for ART [78].

##### Fertility preservation options that male patients should be informed about after completion of cancer-directed therapy

*Microdissection TESE (MD-TESE) for the management of chemotherapy-induced azoospermia* The risk of chemotherapy-induced impairment of spermatogenesis varies with the type and dose of the cytotoxic drug(s) used. If impaired by chemotherapy, spermatogenesis often recovers over time. Therefore, male cancer patients who express an interest in having a child after completion of treatment should be advised to undergo semen analysis. A cohort study in survivors of pediatric cancer did not show an increased risk of congenital anomalies in babies born to fathers who previously received cancer chemotherapy [79]. Hence, standard infertility treatment is empirically indicated for men who previously received cancer treatment, depending on the results of semen analysis. Successful sperm retrieval by MD-TESE has been reported even in some male cancer survivors with persistent azoospermia [80]. The rate of successful sperm retrieval varies with the type of underlying malignancy and the type of cancer treatment provided. The retrieved sperm are usually used for ICSI.

*Hormone replacement therapy for hypogonadotropic hypogonadism* As a late complication of brain radiation, pituitary dysfunction can occur and lead to hypothalamic–pituitary–gonadal axis endocrine disorder associated with impairment of spermatogenesis (hypogonadotropic hypogonadism). If this complication occurs in prepubertal boys, testosterone or human chorionic gonadotropin (hCG) replacement is usually used to induce puberty; however, this approach rarely enables patients to gain spermatogenic function. Instead, hCG/recombinant follicle stimulating hormone (rFSH) replacement during adolescence is recommended for those who may have an interest in having a child in the future [81].

*Interventions to treat erectile/ejaculatory dysfunction* Interventions to treat erectile dysfunction include pharmacological treatment, primarily with a phosphodiesterase type 5 inhibitor; intracavernous injection of prostaglandin E1; and use of a vacuum constriction device or a penile prosthesis [82]. To manage ejaculatory dysfunction, European and American guidelines suggest the use of alpha adrenoceptor agonists, but these drugs have limited efficacy and have not yet gained wide acceptance in Japan. To manage retrograde ejaculation, several reports, primarily from Japan, suggest that tricyclic antidepressants such as amoxapine may be effective [83].

##### Acceptable time to consider pregnancy after completing cancer chemotherapy and optimal timing of sperm harvesting for cryopreservation

As mentioned above, sperm cryopreservation is a fertility preservation method of choice for male cancer patients; in male patients who have an interest in preserving fertility, sperm should be cryopreserved before treatment starts, if the disease status permits. However, cancer-directed therapy may need to be started urgently, which allows limited time for sperm cryopreservation before treatment starts. In this case, patients should be given the opportunity to receive this fertility preservation treatment after a few cycles of treatment or before the start of any treatment that has a high risk for infertility. Of note, the safety of sperm harvested after chemotherapy starts has not been established, and no consensus has been reached regarding the suitability of cryopreserving sperm harvested from men who have initiated cancer chemotherapy. Therefore, before harvesting or cryopreserving sperm, male patients who have started cancer chemotherapy should be fully informed about the benefits and risks of the procedure, and those who have undergone the procedure should be carefully managed and followed up.

A teratogenic drug administered to a male patient may be transferred via the seminal fluid into the body of his female partner. If the female partner becomes pregnant, the drug may exert its teratogenic effect during the early gestational period. Therefore, male patients enrolled in a clinical study of a chemotherapeutic drug with proven teratogenicity are advised to use contraception for 3 months plus 5 times the elimination half-life of the drug after taking the last dose [84, 85]. In practice, it is recommendable to advise male patients to take contraception for a certain duration, although no definitive evidence is currently available regarding the exact time to consider pregnancy after completion of cancer chemotherapy.

### CQ4: how should patients with hereditary cancer be counseled about their fertility?

#### Recommendation 4

4-1. Patients diagnosed with hereditary cancer should be counseled regarding genetic testing and be helped to make treatment decisions, if necessary. (Recommendation Grade B).
4-2. Patients diagnosed with hereditary cancer should be informed that any hereditary cancer is not an indication for prenatal or preimplantation genetic testing in Japan. (Recommendation Grade B).
4-3. Patients diagnosed with hereditary cancer should be informed that little evidence supports the risk of reduced fertility specifically associated with the underlying malignancy. (Recommendation Grade C1)

#### Explanations

CAYA cancer patients and/or their families who express an interest in fertility preservation should be counseled about genetic testing in parallel with counseling about fertility preservation, particularly if their cancer is of early onset or they have a significant family history of cancer, both of which are highly predictive of hereditary cancer [86]. For example, patients who may have hereditary breast ovarian cancer (HBOC) syndrome (a relatively common hereditary cancer syndrome) or Lynch syndrome (a hereditary colon cancer) should be informed that they are candidates for genetic counseling and testing. When counseling patients who may have hereditary cancer, it is essential to consider patients’ family members and relatives. In addition, patients with HBOC syndrome may have an increased risk of other types of cancer, including pancreatic cancer, male breast cancer, and prostate cancer, whereas patients with Lynch syndrome- or Li-Fraumeni syndrome-associated cancers may develop cancer at a variety of primary sites. Therefore, care should be taken not to leave any cancer undiagnosed in patients with such a hereditary cancer. For reference, Lynch syndrome-associated cancers include colon cancer, endometrial cancer, gastric cancer, ovarian cancer, pancreatic cancer, biliary tract cancer, renal pelvic/ureteral cancer, brain tumor (typically, glioblastoma associated with Turcot syndrome), and Muir–Torre syndrome-associated sebaceous gland adenoma and keratoacanthoma. Li–Fraumeni syndrome-associated cancers include soft tissue sarcoma, osteosarcoma, premenopausal breast cancer, brain tumor, and adrenocortical carcinoma. If genetic testing confirms a diagnosis of any such hereditary cancer, patients should be informed about the probability of inheritance of the pathological variant (50%, for example, if the cancer is an autosomal dominant disorder) and the susceptibility of their children to cancer if the pathological variant is inherited (penetrance of cancer in each syndrome). In counseling patients with hereditary cancer, oncologists should receive cooperation from a department or center with expertise in genetic counseling to ensure that patients receive the necessary support from specialists for genetic counseling or therapeutic decision-making. Fertility preservation treatments available for patients with hereditary cancer may include embryo and unfertilized oocyte cryopreservation for future ART. Survivors of hereditary cancers or those with partners who have survived hereditary cancer are candidates for preimplantation genetic testing in some countries/regions (e.g., the UK and several states in the US), but they are not yet candidates for prenatal or preimplantation genetic testing in Japan. An increasing number of oncology clinics are able to perform risk-reducing salpingo-oophorectomy (RRSO) in female patients with HBOC syndrome to minimize their risk of developing ovarian cancer. However, many female patients of reproductive age with this hereditary cancer may wish to preserve fertility and may not agree to undergo RRSO; such patients should be informed that they remain at risk of developing ovarian cancer in the future. Some investigators have suggested worse outcomes of ART in female patients with HBOC syndrome because of their lower ovarian reserve (i.e., lower number of oocytes in the ovaries) [87].

To develop recommendations in an attempt to answer this CQ, we referenced the secondary sources cited in the Introduction—sources that we also referenced for the whole guidelines—and the articles cited in this section, as well as a reference [88].
